# Modification of the Revised National Institute for Occupational Safety and Health (NIOSH) Lifting Equation to Determine the Individual Manual Lifting Risk in Malaysia’s Manufacturing Industry

**DOI:** 10.7759/cureus.57747

**Published:** 2024-04-07

**Authors:** Noor Adillah Dawad, Siti Munira Yasin, Azlan Darus, Ahmad Taufik Jamil, Nyi Nyi Naing

**Affiliations:** 1 Department of Public Health Medicine, Jalan Hospital, Faculty of Medicine, Universiti Teknologi MARA, Sungai Buloh, MYS; 2 Prevention, Medical, and Rehabilitation Division, Social Security Organization, Kuala Lumpur, MYS; 3 Department of Community Medicine, Faculty of Medicine, Universiti Sultan Zainal Abidin, Kuala Terengganu, MYS

**Keywords:** manufacturing industry, ergonomics risk assessment, manual lifting, niosh lifting equation, rnle

## Abstract

Introduction: The National Institute for Occupational Safety and Health (NIOSH) established the Revised NIOSH Lifting Equation (RNLE) for manual lifting risk assessment. The objectives of this study were to determine the characteristics of physical factors using the RNLE and to explore additional factors to RNLE by modifying it to an Individual Lifting Equation (ILE).

Methods: This cross-sectional study was conducted in the manufacturing industry of three states in Malaysia among manual lifting workers. A questionnaire was administered, which comprised the sociodemographic characteristics and Nordic Musculoskeletal Questionnaire (NMQ) assessing low back pain (LBP). The RNLE dataset includes a load constant and six manual lifting variables collected from observational ergonomic risk assessment. The RNLE was modified to ILE by incorporating age, gender, and BMI. The equations’ Lifting Index (LI) computed provides an overall manual lifting risk estimate.

Results: There were 165 participants, with a mean age of 28 years, and 108 (65.5%) were male. Most participants had a BMI within the normal range (60 (36.4%)) or were classified as overweight (54 (32.7%)). The lifting horizontal location showed the highest risk estimates, with the lowest mean multiplier value of 0.55. In contrast, age and BMI had the lowest risk estimates, with mean multiplier values of 0.99 and 0.98, respectively. Among the participants, LI values of one or less, indicating very low risk, were observed in 58 (35.1%) for RNLE and 39 (23.6%) for ILE. Additionally, RNLE and ILE showed figures of 11 (6.7%) and 20 (12.1%), respectively, signifying a very high risk of LI exceeding three.

Conclusion: Studying the lifting factors and equation multipliers from RNLE is critical for evaluating the risk estimates of manual lifting. Exploring the ILE based on individual characteristics is appropriate to support the ergonomic program. Further study is needed to validate the ILE as an accurate screening tool for determining LBP risk estimates.

## Introduction

Low back pain (LBP) is a global health concern. When attributed to occupational factors, it is often associated with occupational physical activity, including manual handling [1]. Improper lifting techniques and inadequate ergonomic conditions can significantly contribute to LBP among workers in various industries, particularly manufacturing [2]. Risk assessment is a fundamental method in ergonomics. It is a field dedicated to optimizing the design and arrangement of workspaces, products, and systems to suit the needs of those who use them better. Ergonomics aims to improve human and environmental interaction to ultimately enhance safety, comfort, efficiency, and overall well-being [3]. In ergonomics, risk assessment involves systematically evaluating and managing potential ergonomic hazards within a workplace or specific task, employing a range of tools and techniques [4]. The Revised National Institute for Occupational Safety and Health (NIOSH) Lifting Equation (RNLE) is an established tool recognized worldwide as the basis for evaluating lifting risk [5].

The NIOSH initially issued the Work Practices Guide (WPG) for Manual Lifting in 1981, which addressed the prevention of musculoskeletal diseases caused by manual lifting [6]. The RNLE was created in 1991 to apply more aspects to lifting tasks, including methods for evaluating asymmetrical lifting tasks, optimal hand-container couplings, and a broader range of work durations. The RNLE variables comprise horizontal and vertical locations, distance of lifting, asymmetry angle, frequency rates, and coupling. The equation provides an empirical Recommended Weight Limit (RWL) calculation method for manual lifting. The ratio of the actual load weight lifted to the RWL is called the Lifting Index (LI), where an LI above one indicates a risk of LBP [7].

The multiplicative property of the RNLE remains supported by additional research. Modifications to the RNLE components are required to estimate risk appropriately. In this context, a new vertical multiplier was modified among Iranian workers [8]. Distance, asymmetric, and coupling multipliers were removed from the equation to predict the performance [9]. Most of these changes have focused on the job’s physical demands. Despite that, a recent study investigated improved risk assessment of RNLE by adding gender and intervertebral disc cross-sectional area multiplier in the equation [10]. The physiological, biomechanical, and psychophysical criteria used to define the equation based on people from the United States (US) may not be accurate for the Malaysian population. Previous studies have shown that the RNLE underpredicts low back injury risk among populations exhibiting diverse anthropometric profiles and variations in pain tolerance, such as those observed in Mexican populations [11]. In addition, the coupling factor in RNLE does not reflect grip strength variability concerning age and gender [12]. Such inconsistencies show that the RNLE may underestimate low-back injury risk with different individual characteristics.

Therefore, developing a lifting equation incorporating individual multipliers is essential to establish precise risk estimations. It has been well-documented that heavy lifting significantly contributes to high compensation claims [1]. The objective findings derived from the lifting equation’s components, which assess risk estimates, can play a pivotal role in enhancing ergonomics programs, especially for groups such as the elderly, female workers, and those with obesity. This aligns to facilitate successful return-to-work programs [13]. Hence, the objectives of the present study among manual lifting workers in Malaysia’s manufacturing industry were twofold: 1) to determine the characteristics of physical factors using the RNLE and 2) to assess the manual lifting risk by modifying the RNLE to Individual Lifting Equation (ILE).

## Materials and methods

Setting and study population

This cross-sectional study was conducted from December 2022 to January 2023 in the manufacturing industry in Peninsular Malaysia. Three states were randomly chosen: Johor, Melaka, and Selangor. Selected companies from each state were approached through their Safety and Health Committees, identified from occupational disease claims made to the Social Security Organization (SOCSO). Purposive sampling targets workers involved in manual lifting jobs within the manufacturing industry, aligning with the study's focus on ergonomic risk assessment using the RNLE. However, selecting participants and companies based solely on RNLE-related criteria might fail to accurately reflect the diversity of manual lifting practices in the broader manufacturing industry. To mitigate this bias, a varied range of companies and participants, fitting the necessary criteria yet diverse in their manufacturing processes, were chosen.

Discussion sessions were conducted with interested companies, and a walkthrough survey was employed to determine work task eligibility for study inclusion. Before commencing the study, the research protocol was approved by the Universiti Teknologi MARA (UiTM) Research Ethics Committee, and written consent was obtained from each participant, ensuring adherence to ethical standards and protecting the rights of all involved individuals. The following criteria were used for selection: 1) jobs involving manual lifting as a regular daily task, 2) jobs with minimal or no unpredictable variations in task characteristics, and 3) jobs that met the requirements for applying the RNLE. Participants were recruited among Malaysian workers who performed manual lifting. Those who worked less than one month, those who were pregnant, those who had underlying spine disease, and those who had a history of back trauma or surgery were excluded.

The questionnaires

This study utilized a set of self-administered questionnaires. Questionnaires included questions on socio-demographic details, job description information, employment experience, and training, and information about the company’s organizational structure (working hours and whether working in shifts). The Nordic Musculoskeletal Questionnaire (NMQ) assesses LBP in the last 12 months and the previous month, a valid and valuable screening tool for musculoskeletal disorders [14]. It has a sensitivity of 100% and a specificity of 88% for chronic or recurring LBP [15]. The Malay translation of the Standardised NMQ (M-SNMQ) revealed that 58.3% of the items had strong kappa agreement values of at least 0.75 [16].

Ergonomics risk assessment

RNLE

The RNLE data were collected by an Ergonomic Trained Person (ETP), certified by the Department of Safety and Health (DOSH), Malaysia, in Posture, Forceful, and Repetition Assessment. This assessment was carried out by a single individual who had also completed an ergonomics course at Universiti Teknologi MARA, which includes a module on the RNLE. Each task was observed for at least 15 minutes during continuous work or for three cycles during non-continuous work. To ensure comprehensive coverage, an assistant maintained the video recorder in a consistent position to capture the entire work process, accompanied by a representative from each company. The assessment was conducted non-intrusively, with direct measurements taken on-site. Distance dimensions were measured using a tape measure, and angles were determined using the adapted modified goniometric platform method [17]. Assessments were conducted close to the worker, focusing on the measuring points for horizontal and vertical locations, and employing the goniometric platform to determine trunk rotation for the asymmetry angle. Video recordings were analyzed to accurately measure the asymmetric angle, especially in cases of fast movement, to ensure precision on the goniometric platform. Frequency and coupling during manual lifting were also assessed, with video analysis further substantiating the findings.

The RNLE consists of one constant and six multipliers. The RWL is calculated as the product of these components as per the following equation: LC x HM x VM x DM x AM x FM x CM = RWL. The RWL is used to compute the LI, which provides relative estimates of the physical stress associated with a specific manual lifting task. The LI is calculated by dividing the load weight representing the total actual weight lifted in kilograms by the RWL. The RNLE multipliers were calculated in accordance with the application manual using formulas from the horizontal location (H), vertical location (V), vertical travel distance (D), and asymmetry angle (A) measurements to compute the Horizontal Multiplier (HM), Vertical Multiplier (VM), Distance Multiplier (DM), and Asymmetric Multiplier (AM), respectively. The manual also includes tables for determining the Frequency Multiplier (FM) and Coupling Multiplier (CM), which require detailed information based on the assessment. Considerations for FM include the average frequency over 15 minutes, vertical location, and duration, while criteria for CM involve coupling classification and vertical location. The following are the detailed components of the equation based on the RNLE application manual [18]. 

i. The LC is 23kg, which refers to the maximum suggested weight for lifting a load under ideal conditions in which all multipliers are equal to one. ii. The HM formula is (25/H) for H measured in centimeters. H is a horizontal location defined as the distance between the mid-point of the line connecting the inner ankle bones and a projected point on the floor just below the mid-point of the hand grasp. iii. The VM is determined using the absolute value of an optimum height of 75cm or deviation from this. For average workers, 75cm is considered the knuckle height. The vertical height of the hands above the floor is the V. The VM formula measured in centimeters is (1-(0.003x(V-75))). iv. The DM formula is (0.82+(4.5/D)) for D measured in centimeters. D is the vertical travel distance of the hands between the vertical location lift’s origin and destination. v. The AM formula is (1−(0.0032A)). Asymmetry is a lift that originates or ends outside the mid-sagittal plane. As per the neutral body position, the sagittal line runs through the middle of the inner ankle bones in the sagittal plane. The A is formed by the asymmetry and mid-sagittal lines when they intersect. vi. The FMs represent lifting frequency (F), which is defined as the average number of lifts per minute measured for 15 minutes, the V, and the duration of continuous lifting, which can be short (one hour or less), long (between more than two hours and eight hours), or moderate (duration values in hours between short and long duration). vii. The CM is based on the lift's coupling classification and V. The types of lift objects for gripping affect the maximum force a worker must apply on the object, and the gripping methods are used to classify coupling as good, fair, or poor.

ILE

The RNLE was modified to ILE by incorporating age, gender, and BMI. The additional individual multiplier calculation is based on [10] as follows. i. The Age Multiplier (AGEM) is implemented to account for projected strength decreases due to aging. For subjects under 40, the age multiplier was 1.0, which decreased by 1% (0.01) each year after that. ii. The Gender Multiplier (GM) is given a score of 1.0 for males. A 2/3 multiplier was applied to female participants. iii. The BMI Multiplier (BMIM) penalizes participants with BMIs exceeding 30. For BMIs less than or equal to 30, it was 1.0. For BMIs more than 30, the BMIM was 30/BMI.

Statistical analysis

Data were analyzed using Stata/SE 18 (StataCorp LLC, College Station, Texas, United States. Descriptive analysis describes the characteristics of manual lifting workers, physical factors in work-lifting, and occupational LBP in Malaysia’s manufacturing industry. The continuous data such as age, working experience, H, V, D of lifting, A, load weight, and multiplier values for each lifting equation component are presented in mean and standard deviations. Categorical data are presented in frequency and percentage, such as LBP, gender, BMI, lifting frequency, coupling, LI, working hours, working shifts, and training. The LBP was identified by aches or pain in the lumbar or buttock areas within a month and in the last 12 months. The manual lifting risk based on LI was identified using the physical and individual factors required to compute multipliers in RNLE and ILE, respectively. The multiplier factors have a value between zero and one. When the multiplier value is lower, the LI is higher, and the risk is more significant. Measurements were taken at their horizontal, vertical, and asymmetry angles to determine LI for the origin and destination points. The measurements that showed higher risk points were chosen. The LI calculated from RNLE and ILE are presented in the results tables. The categorical LI variables were defined as very low (LI ≤ 1.0), low (1.0 < LI ≤ 1.5), moderate (1.5 < LI ≤ 2.0), high (2.0 < LI ≤ 3.0), and very high (LI > 3) [19].

## Results

Out of 175 initially assessed, eight participants did not return their questionnaires, and two were excluded for not meeting the eligibility criteria. A total of 165 participants performing manual lifting tasks from four companies, including automotive, chemical, pharmaceutical, and soap manufacturers, were eligible for the analysis. The characteristics of the manual lifting workers and LBP are presented in Table 1. The mean age of the participants was 28 years. Participants possessed an average of 4.5 years of working experience in their current job. Notably, 94 (57.0%) had not received any safety, health, or ergonomics training. Over the past year and month, 81 (49.1%) and 75 (45.5%) experienced LBP, respectively.

**Table 1:** Characteristics of the manual lifting workers and low back pain (n=165) SD: standard deviation

Variable	Mean (SD)	n (%)
Manual lifting worker		
Age (years)	28.27 (8.11)	
Gender		
Male		108 (65.5)
Female		57 (34.6)
BMI		
< 18.5 (underweight)		23 (13.9)
18.5 – 24.9 (normal)		60 (36.4)
25.0 – 29.9 (overweight)		54 (32.7)
≥ 30.0 (obese)		28 (17.0)
Education level		
Primary		3 (1.8)
Secondary		116 (70.3)
Tertiary		46 (27.9)
Job experience (years)	4.50 (5.36)	
Training		
Yes		71 (43.0)
No		94 (57.0)
Working hours		
≤ 8 hours		27 (16.4)
> 8 hours		138 (83.6)
Work in shift		
Yes		110 (66.7)
No		55 (33.3)
Low back pain		
Presence in the past year		
Yes		81 (49.1)
No		84 (50.9)
Presence in the past month		
Yes		75 (45.5)
No		90 (54.5)

The characteristics of the physical factors include work-lifting factors based on RNLE, load weight, and multiplier components at the origin and destination. The mean load weight was 9.86kg. The mean H and V measured from the origin and destination were relatively similar, at 46.50cm and 49.68cm for Hs and 75.52cm and 79.21cm for Vs, respectively. The mean D was 30.65cm. The mean angle of 60.6° was quite disproportional at the origin compared to its destination point of 34.2°. The coupling was categorized as fair for 150 (90.9%), and most lifts occurred at a rate of less than and equal to 0.2 times per minute for 122 (73.9%). The characteristics of the multipliers in the lifting equation are shown in Table 2 in descending numerical sequence, which indicates increased risk estimates. The horizontal location during lifting was estimated to have the most risk for developing occupational LBP, as indicated by the lowest mean multiplier values at 0.55. In contrast, age and BMI had lower risk estimates, with mean multiplier values of 0.99 and 0.98, respectively.

**Table 2:** Characteristics of the multipliers in the lifting equation (n=165) SD: standard deviation

Variable	Mean (SD)
Age multiplier	0.99 (0.03)
BMI multiplier	0.98 (0.07)
Coupling multiplier	0.97 (0.03)
Distance multiplier	0.96 (0.04)
Vertical origin multiplier	0.92 (0.06)
Vertical destination multiplier	0.91 (0.06)
Gender multiplier	0.89 (0.16)
Frequency multiplier	0.89 (0.09)
Asymmetric destination multiplier	0.89 (0.09)
Asymmetric origin multiplier	0.81 (0.12)
Horizontal origin multiplier	0.59 (0.18)
Horizontal destination multiplier	0.55 (0.18)

Table 3 illustrates the LI according to RNLE and ILE categories that provide the overall risk estimates in manual lifting tasks. LI values of one or less, indicating very low risk, were 58 (35.1%) for RNLE and 39 (23.6%) for ILE. Meanwhile, RNLE and ILE were 11( 6.7%) and 20(12.1%), respectively, signifying a very high risk of LI exceeding three.

**Table 3:** Lifting Index (LI) categories based on the Revised NIOSH Lifting Equation (RNLE) and Individual Lifting Equation (ILE) (n=165) NIOSH: National Institute for Occupational Safety and Health

LI	n (%)	
	RNLE	ILE
LI ≤ 1.0	58 (35.1)	39 (23.6)
1.0 < LI ≤ 1.5	39 (23.6)	42 (25.5)
1.5 < LI ≤ 2.0	31 (18.8)	27 (16.4)
2.0 < LI ≤ 3.0	26 (15.8)	37 (22.4)
LI > 3.0	11 (6.7)	20 (12.1)

## Discussion

This study aimed to modify the RNLE into the ILE to determine individual manual lifting risks with a specific focus on age, gender, and BMI. The descriptive findings indicate a notable difference in manual lifting risk, as determined by the LI, between the RNLE and ILE. Particularly, the difference is prominent in the very low-risk category, where the LI calculated using the RNLE predominantly fell within this category. In contrast, the LI findings derived from the ILE indicated higher risk estimates, especially in the high and very high-risk categories. This suggests that the ILE may provide a more nuanced assessment of manual lifting risks, identifying higher risk levels that the RNLE may have overlooked. Further investigation into the factors contributing to these discrepancies could offer valuable insights into the applicability of both assessment methods. Validation of the modified lifting equation from this study as an accurate screening tool for determining manual lifting risk estimates, such as ILE, is necessary. Details regarding the validation part of the modified lifting equation will be in a separate review.

Risk assessment during manual lifting is essential in implementing effective measures to support an ergonomic program. By identifying and addressing potential risks early on, adjustments can be made to work tasks to reduce the likelihood of injuries. For instance, studies have shown that making appropriate adjustments based on risk assessment can significantly reduce the LI from high-risk levels [20]. Moreover, through risk identification, critical risk factors, such as physical factors outlined in the NIOSH Lifting Equation, can be identified [21]. Most studies reported that the standard variables contributing to the high-risk estimate were V and H [22,23]. This study also found that H during lifting posed the highest risk during manual lifting, based on the lowest multiplier value. This finding is consistent with the conclusions of another study, which similarly identified that maximum horizontal distance is associated with higher odds of having LBP [24].

The variables of the NIOSH Lifting Equation have been determined through expert consensus and a comprehensive literature review [7]. In modification of the lifting equation, choosing variables for the prediction model is often the most crucial step. While a previous study [10], Barim, et al. formed the foundation for modifying the RNLE in this study by assessing the inclusion of age, gender, BMI, and the intervertebral disc cross-sectional area multiplier, our study focused solely on integrating all individual factors except for the intervertebral disc cross-sectional area multiplier. This decision was made due to practical constraints regarding the availability of findings related to the intervertebral disc cross-sectional area multiplier. Moreover, the principle of parsimony is fundamental in prediction model selection, favoring simpler models over complex ones. Balancing goodness-of-fit with model complexity is essential to prevent overfitting [25]. The collected datasets for this study demonstrate completeness; all essential variables and pertinent information have been diligently gathered, providing a comprehensive and reliable basis for analysis and interpretation.

The study encompassed a wide range of ages and BMIs, with 57 (35%) of the participants being female workers. The occupational factors among manual lifting workers revealed several key findings. The mean working experience among participants was five years, yet half reported receiving no safety, health, or ergonomics training. Studies have shown that ergonomics training effectively enhances workers’ knowledge, self-efficacy, and practices in preventing LBP [26]. This disparity between experience and training underscores the need for further action to address the potential gaps. Additionally, it warrants future research to examine the impact of safety, health, and ergonomics training on preventing LBP. Many manual lifting workers in the manufacturing industry reported being engaged in shift work, often requiring periods exceeding eight hours daily. It is important to note the details of these extended work hours, as other studies have reported that individuals working over 50 hours per week or more than 10 hours per day are at a heightened risk of encountering occupational health issues [27]. Over the past year, nearly half of the participants, precisely 81 (49.1%), reported experiencing LBP. This suggests that LBP is a persistent issue among workers in this occupational setting, potentially impacting their daily activities and overall well-being. Furthermore, within the more recent timeframe of the past month, a substantial proportion of participants, 75 (45.5%), reported experiencing LBP. This indicates that the prevalence of LBP remains consistent over time, with a notable percentage of workers experiencing symptoms on a recurring basis.

The RNLE, an observational ergonomics risk assessment method, is among the techniques used to evaluate manual lifting risks alongside self-reports and direct measurements. Observational techniques are widely favored in workplaces for their cost-effectiveness and practicality. However, direct measurements generally offer more reliable data than observations or subjective evaluations [28]. Various methodologies exist for assessing exposure to work-related musculoskeletal disorder risks, and the choice among them depends on specific application and study objectives. Recently, researchers have advanced ergonomic risk assessment techniques by replacing human observers with instrumental approaches based on wearable sensors [29]. In this study, the modified goniometric platform method was manually utilized as the angle measurement technique. However, it is essential for future studies to consider employing a robust computer vision-based method for estimating the load asymmetry angle defined in the RNLE [30].

The criteria for enrolling companies were deliberately designed to be inclusive, aiming to encompass a broad spectrum of participants within the manufacturing industry in Malaysia. This approach ensures that the study’s findings reasonably represent the diverse population in this sector. This study does have its set of limitations. According to the RNLE, one notable restriction is that jobs involving pushing, pulling, or carrying tasks should not be analyzed using this lifting equation [18]. This limitation is quite restrictive, significantly reducing the number of jobs that can be analyzed using this method. As a result, many jobs involving pushing, pulling, or carrying tasks often intertwined with lifting and lowering activities were excluded from the analysis. The present study selected manual lifting tasks based on their primary lifting or lowering components, following the guidelines outlined in the RNLE application manual [18]. However, it is essential to note that most of the selected subjects were also involved in carrying. Carrying tasks, in particular, were commonly performed with lifting and lowering activities. The calculations assume negligible other manual material handling activities. This intertwining of tasks highlights a potential limitation of the NIOSH Lifting Equation, as it does not account for the combined effects of carrying tasks alongside lifting and lowering demands.

Secondly, a limitation concerns the study's findings, which may be specific to the time and conditions under which it was conducted. Changes in workplace practices, ergonomic interventions, or health policies over time could influence the relevance and applicability of the study’s results in the future. Thirdly, participation in the study was voluntary, and workers who agreed to participate may differ in some characteristics from those who chose not to participate. This self-selection bias could impact the representativeness of the study sample. Fourthly, having a single observer, especially one well-trained in RNLE, ensures consistency in data collection methods and reduces the variability that might arise from different interpretation styles. However, this approach may also introduce bias, as the assessment is confined to the observer’s viewpoint and expertise. This requires multiple observers to be equally trained to ensure consistency in their assessments, which can be a challenging and resource-intensive process. Lastly, the descriptive findings from a cross-sectional study design offer valuable insights into the prevalence and distribution of ergonomic risk factors. However, they may lack the depth necessary to establish causal relationships. Consequently, the study’s descriptive nature limits its ability to validate differences between the RNLE and the ILE, hindering conclusive comparisons between the two approaches.

This study represents a preliminary step toward understanding the differential between the RNLE and ILE in assessing manual lifting risks. While our findings provide valuable insights, particularly highlighting the increased risk identification capability of the ILE compared to the RNLE, several areas warrant further investigation to enhance our understanding and application of ergonomic risk assessments. The future research directions outlined below aim to build upon the groundwork laid by this study, addressing the gaps identified and offering pathways for comprehensive ergonomic risk management.

Longitudinal studies for causal inference

Longitudinal designs are essential to track changes over time, providing invaluable insights into how manual lifting practices directly contribute to the development of musculoskeletal disorders (MSDs), with a particular focus on LBP.

Diverse populations and work settings

Expanding research to encompass more diverse populations, cultural contexts, various work settings, and non-manufacturing environments will enhance the generalizability of the findings.

Interobserver reliability 

Investigating the impact of single versus multiple observers on the outcomes of RNLE assessments could refine our approach to data accuracy, fostering methodologies that combine consistency with a comprehensive perspective. Future research should prioritize the training of additional observers in the RNLE methodology to ensure enhanced interobserver reliability during assessments.

Objective measurement tools

Our reliance on self-reported data and manual measurements points to a significant area for improvement. The integration of wearable sensors and computer vision techniques promises a leap toward precision, offering real-time, objective data that could revolutionize risk assessments.

Integration with comprehensive ergonomic management systems

Exploring the integration of the ILE within broader ergonomic management systems is warranted. Validating the modified RNLE equation is a necessary step. Future research should focus on how individualized risk assessments can be systematically incorporated into comprehensive ergonomic programs covering manual lifting. Such an approach can potentially promote a holistic workplace health and safety culture.

## Conclusions

This study contributes to industry knowledge and practices concerning assessing risk estimates associated with manual lifting by studying the RNLE. Exploring the ILE based on individual characteristics is appropriate to support the ergonomic program. Moving forward, addressing ergonomic concerns requires the implementation of a comprehensive plan within an ergonomic management system. Especially given the aging and female workforce as well as the rising prevalence of obesity, further study is needed to validate the ILE as an accurate screening tool for determining LBP risk estimates.
